# Harnessing the Forgotten Triangles of the Neck for Lingual Artery Ligation in Glossectomies: A Technical Report

**DOI:** 10.7759/cureus.43836

**Published:** 2023-08-21

**Authors:** Sandeep Ghosh, Bonny Joseph, Priyanko Chakraborty, Soumya Singh, Epshita Das

**Affiliations:** 1 Surgical Oncology, Chirayu Medical College and Hospital, Bhopal, IND; 2 Surgical Oncology, Malabar Cancer Center, Thalassery, IND; 3 Otolaryngology - Head and Neck Surgery, Institute of Medical Sciences, Banaras Hindu University, Varanasi, IND; 4 Anaesthesiology, Chirayu Medical College and Hospital, Bhopal, IND; 5 Pathology and Laboratory Medicine, Vardhman Mahavir Medical College and Safdarjung Hospital, New Delhi, IND

**Keywords:** tongue cancer, head and neck oncology, lingual artery, glossectomy, lesser's triangle, pirogoffs's triangle, forgotten triangles of neck, neck triangles

## Abstract

Various geometric structures, such as numerous triangles, are prevalent in the neck. Despite the fact that many of the neck's anatomical triangles have been well documented, others have been almost forgotten about. The repertoire of head and neck cancer surgeons should include an in-depth understanding of these forgotten triangles. This article seeks to contribute valuable information to the existing scientific literature by shedding light on these neglected triangles, which have significant surgical relevance. In this technical report, we provide a detailed description of a technique that employs these neglected triangles to ligate the lingual artery during glossectomies for tongue cancer.

## Introduction

The neck is a region abundant in geometric structures, such as numerous triangles. While many of the neck's anatomical triangles have been well documented, others have been overlooked and almost forgotten. Each side of the neck is anatomically divided into two principal, well-defined triangles known as the anterior triangle and the posterior triangle [1]. Lateral boundaries of the anterior triangle are defined by the anterior border of the sternocleidomastoid muscle, medial boundaries by the midline, and superior boundaries by the lower border of the mandible. Similarly, the posterior triangle is delineated by the posterior border of the sternocleidomastoid muscle on the anteromedial side, the anterior border of the trapezius muscle on the posterolateral side, and the clavicle on the inferior side [2].

The deep fascia of the neck envelops both of these triangular regions, and it is only upon removal of this fascia that the secondary triangles of each side of the neck become visible. Within the anterior triangle are three coupled triangles known as the carotid (superior carotid), submandibular (submaxillary or digastric), and muscular (inferior carotid) triangles. In addition, an unpaired triangle, known as the submental triangle, is located beneath the chin between the anterior bellies of the digastric muscle [2].

Within the submandibular triangle are two lesser known, almost forgotten, anatomical triangles: Lesser's triangle, named after the eminent Polish-born German surgeon Ladislaus Leo Lesser (1846-1925), and the Pirogoff's triangle, named after the respected Russian surgeon Nikolai I. Pirogoff (1810-1881) [1]. Despite their significance in the identification and ligation of the lingual artery as well as the identification of the hypoglossal nerve, these triangles are seldom discussed in medical literature [2].

This article seeks to contribute valuable information to the existing scientific literature by shedding light on these neglected triangles, which have significant surgical relevance. In this technical report, we provide a detailed description of a technique that employs these neglected triangles to ligate the lingual artery during glossectomies for tongue cancer.

## Technical report

Before conducting glossectomies for carcinoma of the tongue, we frequently employ either Lesser's triangle or its posterior continuation, known as Pirogoff's triangle, to identify and ligate the lingual artery. Both of these triangles are situated within the clearly defined submandibular triangle.

The superior boundary of Lesser's triangle is formed by the hypoglossal nerve, while the anterior belly of the digastric muscle and the intermediate tendon of the digastric muscle constitute the inferior boundary. The posterior boundary is demarcated by the posterior margin of the mylohyoid muscle at the intermediate tendon of the digastric muscle [1].

Pirogoff's triangle, on the other hand, is bounded superiorly by the hypoglossal nerve and inferiorly by the intermediate tendon of the digastric muscle. The anterior border is determined by the posterior edge of the mylohyoid muscle. The floors of both of these anatomical triangles are composed of the hyoglossus muscle [1].

Before proceeding with hemiglossectomies, subtotal glossectomies, or total glossectomies to treat tongue malignancies, our head and neck oncology practice utilizes these lesser-known neck triangles for lingual artery ligation.

**Figure 1:**
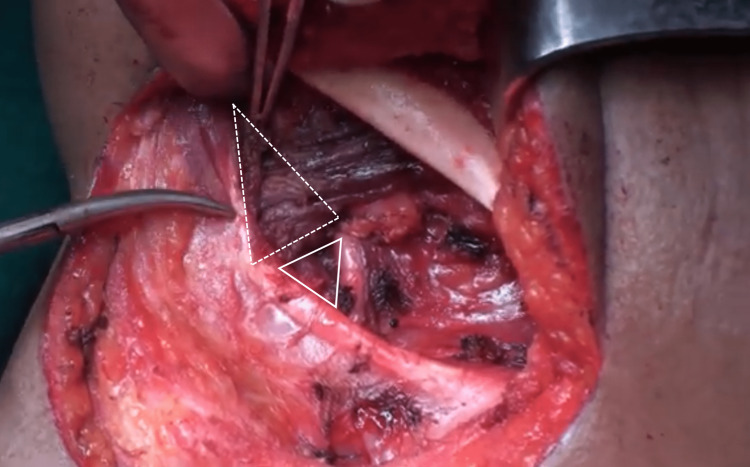
The submandibular triangle after the submandibular gland and Level IB lymph nodes have been removed. The area within the outlines of the solid triangle represents Pirogoff's triangle, while the area within the dashed triangle represents Lesser's triangle under the mylohyoid muscle.

**Figure 2:**
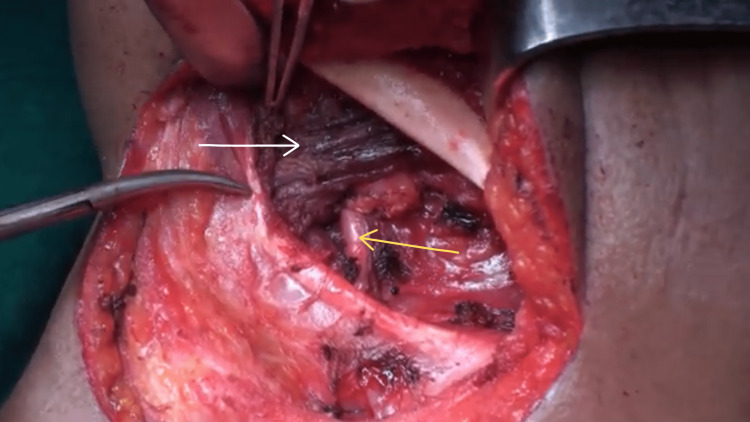
The white arrow represents the mylohyoid muscle, while the yellow arrow shows the hypoglossal nerve.

During neck dissection, the patient lies supine with the neck extended and supported by a shoulder roll, while the head is turned to the opposite side. We prefer a single transverse neck crease incision, typically made four fingers below the lower border of the mandible. Subplatysmal flaps are meticulously elevated. The Level IA and Level IB lymph nodes, along with the submandibular gland, are then removed. The focus then shifts to the identification of Lesser's and Pirogoff's triangles (Figures 1, 2).

**Figure 3:**
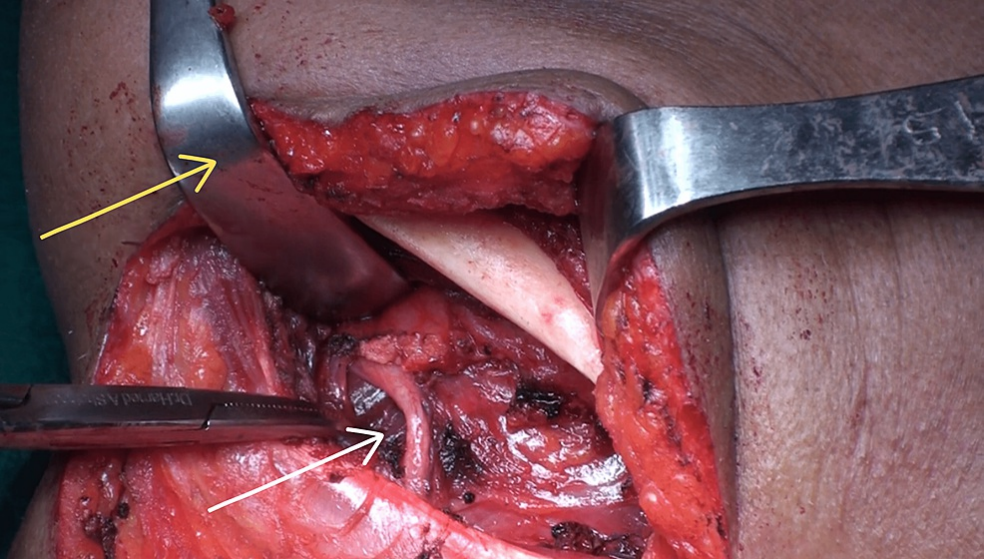
The yellow arrow identifies the Langenbeck retractor, which retracts the mylohyoid muscle, whereas the white arrow points to the hyoglossus muscle, which forms the base of Pirogoff's triangle.

**Figure 4:**
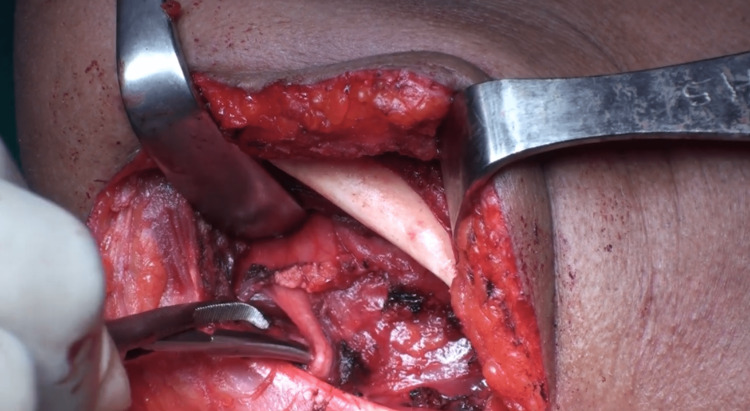
Dissection of the hyoglossus muscle at Pirogoff's triangle, just beneath the hypoglossal nerve.

The mylohyoid muscle is then retracted with a Langenbeck retractor (Figure 3), and the hyoglossus muscle is dissected just below the hypoglossal nerve with Mixter forceps (Figure 4).

**Figure 5:**
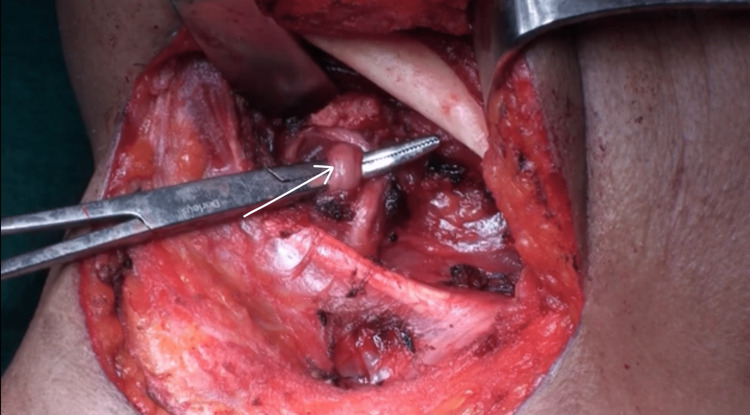
Identification and preparation for ligation of the lingual artery. The white arrow indicates the lingual artery.

This technique permits immediate visualization of the lingual artery, which lies 3 mm inferior to the hypoglossal nerve. It is then looped (Figure 5) and tied with 2-0 silk sutures.

For hemiglossectomies, the lingual artery is typically ligated on the ipsilateral side, whereas bilateral ligation is performed for subtotal or total glossectomies. Ligating the lingual artery within these triangles prior to surgically excising the tongue cancer yields several advantages. It facilitates a bloodless and clean surgical field during the wide local excision, enabling us to achieve a negative surgical resection margin of more than 10 mm without encountering complications. Furthermore, this technique substantially reduces the operating time required for glossectomies. Following the surgical excision of Level IB lymph nodes and the submandibular gland, lingual artery ligation is typically performed in less than five minutes.

## Discussion

The lingual artery is an anteromedial branch of the external carotid artery that supplies the oral cavity, particularly the tongue and floor of the mouth, with blood. In its initial portion, the artery passes through Beclard's triangle, a subset of the much larger carotid triangle. In its second portion, the artery lies deep beneath the hyoglossus, digastric, and stylohyoid muscles. At this juncture, it is described that the vessel lies within both Lesser's and Pirogoff's triangles. In its third portion, the vessel bends abruptly upward at the anterior border of the hyoglossus and divides into the sublingual and deep lingual arteries, which may connect with other vessels beneath the frenulum of the tongue. Nonetheless, this trajectory is subject to substantial variation [3].

Pirogoff's triangle has been advocated as the optimal access point to the lingual artery in cases of severe lingual hemorrhage [4]. In addition, it has been investigated as a viable alternative when searching for a suitable recipient artery in the neck for free vascularized tissue transfer in reconstructive procedures addressing defects in the maxillofacial region [5]. Another potential use of Pirogoff's triangle has been elucidated in this technical report.

During glossectomies, the conventional approach for lingual artery ligation involves performing it during resection when encountered intraorally. However, the tongue, being a highly vascular organ, can lead to significant bleeding during resection, resulting in a bloody surgical field. This excessive bleeding may occasionally compromise the surgical resection margins, leading to suboptimal oncological outcomes for the patient.

Kubic et al. [6] conducted a noteworthy study on the efficacy of transcervical arterial ligation in patients undergoing transoral robotic-assisted surgery (TORS) for the base of tongue tumors. With this intervention, their findings demonstrated a significant protective effect against postoperative major or severe bleeding (p < .04). Only one (1.3%) of the 74 patients who underwent concurrent arterial ligation and TORS encountered postoperative major or severe hemorrhage. The subset of patients who did not undergo arterial ligation had a significantly higher rate of major or severe hemorrhage (7.8%). In addition, the need for reoperation was significantly reduced in the cohort that underwent arterial ligation. They concluded that transcervical arterial ligation has the potential to substantially reduce the severity of postoperative bleeding events. In light of these findings, they suggest transcervical arterial ligation be considered in all cases of TORS involving concurrent neck dissection, highlighting its potential to improve patient outcomes and reduce the need for subsequent surgical interventions. Mandal et al. [7] investigated the effects of prophylactic transcervical arterial ligation on the occurrence of post-operative hemorrhage in a similar study. Prophylactic arterial ligation was administered to 33 of the 224 patients undergoing TORS at their prestigious institution. Their findings shed light on the significance of intraoperative ligation of main vessels in reducing the severity of postoperative bleeding incidents. Notably, none of the five patients who experienced a major postoperative hemorrhage had undertaken the preventative ligation procedure. In response to these compelling observations, their institution has adopted a paradigm shift, routinely administering prophylactic transcervical arterial ligation during neck dissection in the majority of cases.

Baliga and Jaisani [8] have provided a comprehensive justification for the elective extraoral ligation of the lingual artery in the context of the management of oral tongue squamous cell carcinoma. Typically, their procedure involves ligating the lingual artery at its site of divergence from the carotid system, specifically at the level of the hyoid bone. Before undertaking tongue resection, the authors meticulously delineate the numerous benefits of pre-emptive lingual artery ligation in the neck. This proactive measure has demonstrated significant benefits, most notably a significant decrease in intraoperative blood loss. In addition, this strategy reduces the need for blood and blood product transfusions.

In this technical report, the extraoral, transcervical ligation of the lingual artery at Lesser's or Pirogoff's triangle prior to tongue resection is described. This approach offers several advantages, including reduced blood loss, resulting in a clean and bloodless surgical field that does not compromise the surgical resection margin. Additionally, this method has the potential to decrease the glossectomy operating time.

However, it is important to note that a larger randomized controlled trial is necessary to establish the efficacy of this approach. A head-to-head comparison of these two techniques of lingual artery ligation during glossectomies for tongue cancer is required to further highlight the advantages of utilizing the forgotten triangles of the neck.

## Conclusions

The repertoire of head and neck cancer surgeons should include an in-depth understanding of these forgotten triangles. This technical report is intended to reduce surgical morbidity and improve the efficacy of surgical dissections when performing glossectomies. In conclusion, lingual artery ligation at Lesser's or Pirogoff's triangle prior to tongue resection demonstrates promise for enhancing surgical outcomes during glossectomies. Further research through a robust clinical trial will help validate its benefits and establish it as a potential alternative to the conventional method.
